# Men’s knowledge and awareness of maternal, neonatal and child health care in rural Bangladesh: a comparative cross sectional study

**DOI:** 10.1186/1742-4755-9-18

**Published:** 2012-09-03

**Authors:** Hashima E Nasreen, Margaret Leppard, Mahfuz Al Mamun, Masuma Billah, Sabuj Kanti Mistry, Mosiur Rahman, Peter Nicholls

**Affiliations:** 1 Research and Evaluation Division, BRAC Centre, Dhaka, Bangladesh; 2 University of Aberdeen, Aberdeen, Scotland, UK; 3 BRAC Health Programme, BRAC Centre, Dhaka, Bangladesh; 4 University of Southampton, Highfield, Southampton, UK

**Keywords:** Men’s knowledge, Improving Maternal, Neonatal and Child Survival (IMNCS), Women’s reproductive health, Essential newborn care, Bangladesh

## Abstract

**Background:**

The status of men’s knowledge of and awareness to maternal, neonatal and child health care are largely unknown in Bangladesh and the effect of community focused interventions in improving men’s knowledge is largely unexplored. This study identifies the extent of men’s knowledge and awareness on maternal, neonatal and child health issues between intervention and control groups.

**Methods:**

This cross sectional comparative study was carried out in six rural districts of Bangladesh in 2008. BRAC health programme operates ‘improving maternal, neonatal and child survival’ intervention in four of the above-mentioned six districts. The intervention comprises a number of components including improving awareness of family planning, identification of pregnancy, providing antenatal, delivery and postnatal care, newborn care, under-5 child healthcare, referral of complications and improving clinical management in health facilities. In addition, communities are empowered through social mobilization and advocacy on best practices in maternal, neonatal and child health. Three groups were identified: intervention (2 years exposure); transitional (6 months exposure) and control. Data were collected by interviewing 7,200 men using a structured questionnaire.

**Results:**

Men prefer to gather in informal sites to interact socially. Overall men’s knowledge on maternal care was higher in intervention than control groups, for example, advice on tetanus injection should be given during antenatal care (intervention = 50%, control = 7%). There were low levels of knowledge about birth preparedness (buying delivery kit = 18%, arranging emergency transport = 13%) and newborn care (wrapping = 25%, cord cutting with sterile blade = 36%, cord tying with sterile thread = 11%) in the intervention. Men reported joint decision-making for delivery care relatively frequently (intervention = 66%, control = 46%, p < 0.001).

**Conclusion:**

Improvement in men’s knowledge in intervention district is likely. Emphasis of behaviour change communications messages should be placed on birth preparedness for clean delivery and referral and on newborn care. These messages may be best directed to men by targeting informal meeting places like market places and tea stalls.

## Background

Male partner involvement in women's sexual and reproductive health as well as maternal and child health care has recently attracted considerable attention. The International Conference on Population and Development (ICPD) in Cairo, 1994 [1] and the 4^th^ World Conference on Women in Beijing [2] drew attention to women’s health and the need to have men more involved in the promotion of sexual and reproductive health. Although the notion of ‘men as partners’ was contested in Cairo by some of the women’s movements [3], both conferences emphasized men’s shared responsibility and active partnership in sexual and reproductive health and promotion of gender equality [1,2].

Changing and improving the way men are involved in reproductive health problems can also have positive impact on women’s, men’s and children’s health [4,5]. Evidence also shows that men can prevent unintended pregnancies, reduce unmet need for family planning (FP), foster safe motherhood and practice responsible fatherhood [6]. In the USA, partner involvement in pregnancy has increased antenatal care 1.5 times [7]. Even in India, a maternity care model that encouraged husband’s participation in their wives’ antenatal and postnatal care found positive changes in knowledge, gender roles and decision-making [8]. In addition, demographic and health surveys in five Latin American countries (Bolivia, Peru, Colombia, Haiti and Nicaragua) indicated that positive couple interaction is associated with improved health outcome for children [9].

Previous studies suggest various ways in which men mediate and restrict women’s access to health care services including men’s decision-making authority [10-16], their influence over material resources including financial resources [10,14], low level of basic knowledge in any of maternal and child health care issues [11,12], and cultural barriers that pose restrictions on women’s movement and exclude men from taking part in women’s health [17]. In many cultures, men, older women and families make decisions to take contraceptives, when and where to seek treatment and the type of services to use, whether to pay for skilled assistance or transportation to a hospital, that affect women’s sexual and reproductive health and contribute to high incidences of reproductive disease, disability and death [9,11,15].

In Bangladesh, predominantly a patriarchal society, women’s access to social, economic, politico-legal and health care institutions is largely mediated by men. Within the household and in the public sphere, men control women’s sexuality, their choice of marriage partner, their access to labour and other markets and their income and assets [18,19]. This affects women’s health and health-seeking behaviour in several ways, firstly, by controlling behaviours and decision-making authority of husbands and elderly members [20-22], secondly, through neglect and low prioritization of women’s health issues [23,24] and finally, because of cultural beliefs that consider morbidity during pregnancy a normal consequence of pregnancy [25]. Other prominent barriers to male involvement in maternal health are social stigma derived from notions of bad fate (awful happening linked with women’s luck) associated with an abnormal pregnancy or delivery; shyness and embarrassment at having to deal with ‘women’s matters’ publicly; and job responsibilities [26-28].

With the Millennium Development Goals (MDG) of reducing maternal, neonatal and child mortality in Bangladesh in mind, BRAC has initiated a large community-based programme to reduce maternal, neonatal and child mortality in 2005 in Nilphamari and has taken a decision to scale up in three new districts (Rangpur, Gaibandha and Mymensingh) in 2008. There is limited literature to inform our understanding of what happens at a micro level in terms of men’s knowledge and practice in relation to antenatal, delivery and neonatal care. To address this shortcoming, this study explores the knowledge of men on maternal and child health issues, their awareness of their wives’ practices and the preferred means of decision-making.

The objective of the study is to compare men’s knowledge and awareness of their wives’ practices, and the preferred means of decision-making on maternal, neonatal and child health issues between intervention and control districts.

## Methods

### Study setting

This cross-sectional comparative study was conducted in six northern rural districts of Bangladesh. These districts are broadly representative of rural Bangladesh, where agriculture is the main occupation for more than 90% of people, 60% do not know how to read and write, 40% are below the poverty line, and more than 90% of women are housewives.

BRAC executes its core development initiatives i.e. microfinance, education, community empowerment, human rights and legal services (HRLS), water, sanitation and hygiene (WASH), and health in all six study districts. In addition to this, BRAC health programme (BHP) operates ‘improving maternal, neonatal and child survival’ (IMNCS) project in four of the above-mentioned six districts. Hence, our study areas were divided into three groups based on the existence or duration of the IMNCS intervention. As the IMNCS project was started in August 2005 in Nilphamari, we classified this district as the ‘intervention’. In Rangpur, Gaibandha and Mymensingh, the project was initiated in February 2008, just six months before the survey period, so we expected little effect from the IMNCS activities. This was termed as the ‘transition’ group. Naogaon and Netrokona were our control areas as they were devoid of IMNCS activities and had geographical and cultural similarities with the other districts.

BRAC’s IMNCS intervention comprises a number of components aiming to reduce maternal, neonatal and child mortality and morbidity, particularly among the poor and socially excluded population. The components include improving awareness of FP, identification of pregnancy, providing antenatal, delivery and postnatal care, essential newborn care, referral of complications and improving clinical management in health facilities [29].

Active involvement of the men/husbands needs to be ensured as they are usually the decision-makers in the families. Therefore, some activities were designed to improve their role in maternal, neonatal and child health (MNCH) in the community. As part of the IMNCS intervention, during the last trimester of pregnancy (possibly at the seventh month), birth planning (to determine place of delivery, attendant at delivery, save money and arrange transport for emergency referral) for the pregnant woman is done by IMNCS programme organizers in the presence of her husband and other members of the family to motivate them to follow the steps for a safer delivery. In addition, MNCH committees consisting of 9–11 members from accepted local elites and influential persons (e.g., school teacher, religious leader, village doctor etc.) are formed by the programme organizers. Important MNCH issues are discussed in MNCH committee meetings organized by programme organizers at regular interval [30]. The committees monitor and facilitate provision of MNCH services at community level, arrange community financing, support referral of complicated cases to health facilities, arrange transport for referral and audit deaths. Orientation of Imams (religious leaders) and village doctors (alternative health care providers) and union advocacy meetings were also devised to improve the involvement of men/husbands in MNCH care services.

### Study population

This study included male respondents who were husbands of women interviewed as part of a female baseline survey conducted in 2008 [29]. Two groups were sampled: men whose wives had a live birth, a still birth, an intrauterine death, menstrual regulation or abortion in the year preceding the survey; or whose wives had a live child aged 12–59 months at the time of survey.

### Sampling

As mentioned earlier, respondents for this survey were husbands of women randomly selected for 2008 female baseline survey. Therefore, the required sample size for this study was same as that of the female baseline survey 2008 [29]. Hence, to obtain 80% power and a 5% level of significance, and assuming a design effect of 1.5 and non-response rate of 3%, the estimated sample size was 1,200 men (600 in each of the two groups) in each district [29]. This yielded a total of 4,800 men for four intervention and 2,400 men for two control districts.

### Survey instrument

Structured questionnaire was used to collect socio-demographic information, men’s knowledge on reproductive history of women, maternity care, newborn care, and newborn and under-5 childhood illnesses. Information on men’s awareness of their wives’ use of FP methods, taking maternity and newborn care, and care during newborn and under-5 childhood illnesses was also collected. We also collected information on who took the decision regarding the use of FP and receiving maternity care of their wives.

### Data collection

The questionnaire was constructed based on the MNCH baseline survey 2008 questionnaire [29]. It was pre-tested and finalized in October 2008 in Gazipur (a non-study area) by three trained and educated male interviewers. Thirty-six male enumerators and six monitors were recruited and trained for 10 days. They subsequently listed households and collected data from October 2008 to January 2009. Of the 7,200 respondents selected for the survey, 5,547 were interviewed. The overall response rate was 77%. To ensure quality of data, a four-layered monitoring system was developed. The first layer was composed of team members who monitored each other’s activities. Their work in turn was cross-checked by the six rotating monitors who interchanged their places at intervals. Field activities were controlled and monitored by a field supervisor. The lead researchers from the central office monitored field activities through frequent visits.

### Data analysis

The collected data were cleaned, stored and analyzed using SPSS version 11.5. The analysis involved calculation of summary statistics used in comparing grouped districts. Independent t-tests were used to assess differences between means. The chi-squared tests were used to assess categorical differences between grouped districts.

### Ethical approval

Ethical approval was obtained from the Bangladesh Medical Research Council (BMRC) which reviewed the proposal, questionnaire and consent form before providing clearance. In addition, informed consent was taken from the participants before every interview. Confidentiality was maintained by removing all identifiers of the respondents during data entry.

## Results

This section includes the comparison between intervention and control areas (and not the transitional areas). A paragraph describing the findings of the transitional areas is presented at the end of the results section.

### Background characteristics of respondents

Education and literacy levels were similar across all areas. The mean age of respondents was significantly lower in the intervention area compared to the other two (Table 1).

**Table 1:** Background characteristics

	**Intervention**	**Transition**	**Control**	**p**	**p**	**p**
	**(1)**	**(2)**	**(3)**	**1 vs. 2**	**1 vs. 3**	**2 vs. 3**
N	959	2609	1979			
Mean age (SD)	32.1(±.64)	33.72(±7.4)	33.37(±7.359)	.000	.000	.115
Literacy (Can read & write) (%)	43.8	43.4	44.0	.844	.932	.718
Mean years of schooling	3.69(±4.10)	3.63(±4.31)	3.57(±4.12)	.699	.491	.688
Educational status (%)						
No education	42.4	48.4	46.8	.067	0.003	.000
Primary incomplete	16.8	11.0	11.7			
Primary	13.0	11.2	13.1			
Secondary incomplete	17.3	16.8	17.9			
Secondary or higher	10.3	11.6	9.9			
Don’t know	0.1	0.9	0.5			
Main occupation (%)						
Farming	27.6	25.2	32.3	.006	.014	.000
Day labour	31.5	27.8	30.1			
Service	3.6	5.1	3.5			
Business (small and big)	17.2	19.2	16.3			
Skilled labour	4.3	6.6	3.7			
Driver (rickshaw/van)	11.2	10.5	7.9			
Others (unemployed, village doctor etc.)	4.6	5.7	6.1			

### Social involvement

In the intervention area, 11.7% of men compared to 20.3% in control districts were members of clubs, committees or *samity*. Microfinance, religious and sports clubs were the most frequented. Market places or tea stalls were more popular forms of social interaction with 99.2% of men in intervention and 94.1% in control areas using these as informal meeting places with 25 to 30 hours every month spent in these places. Entertainment, political, developmental, sports and religious issues were the main topics of their conversation (data not shown).

### Men’s knowledge on selected maternal, neonatal and child health issues

#### Age at marriage and conception

The legal age of marriage for women is 18 years in Bangladesh. More than 90% of the respondents recognized it correctly. Seven in every ten respondents said that the age at first conception should be at least 20 years irrespective of study setting (Table 2).

**Table 2:** Men’s knowledge on maternal and neonatal care

	**Intervention**	**Transition**	**Control**	**p**	**p**	**p**
	**(1)**	**(2)**	**(3)**	**1 vs. 2**	**1 vs. 3**	**2 vs. 3**
N	959	2609	1979			
Age when girls should get married (≥ 18 years)	93.7	93.5	91.4	.020	.958	.068
Age when girls should conceive (≥ 20 years)	71.8	72.0	79.9	.000	.081	.000
N	411	1032	793			
Knows about ANC	99.3	95.2	98.5	.000	0.062	.001
Services that a woman should receive*						
Advice on Tetanus Toxoid (TT) vaccination	49.6	27.3	7.1			
Advice on dietary intake	85.4	41.8	63.7			
Advice on resting	75.7	51.3	61.8			
Advice on Iron folic acid intake	58.2	45.2	46.0			
Advice on newborn care	1.7	2.6	0.4			
Advice on family planning	1.0	2.6	3.9			
Advice on complications	0.7	3.4	4.3			
Advice on birth preparedness	1.0	3.9	0.6			
Know phone number of health worker	6.8	1.6	0.8			
Advice on not doing any heavy work	76.2	46.2	73.1			
Pulse examination	41.6	21.3	25.9			
Blood pressure	64.5	16.6	31.8			
Weight measurement	52.8	23.4	14.9			
Height measurement	16.1	2.2	2.0			
Anemia	15.8	4.6	11.7			
Blood test	23.4	24.0	36.6			
Urine test	26.3	30.5	38.2			
Abdominal examination	59.6	29.4	55.9			
Foetal heart beat	4.1	.9	1.5			
Ultrasonogram	11.4	21.1	23.2			
Don’t know	2.9	17.5	2.4			
Birth preparedness						
Determine attendant at delivery	84.7	62.7	79.7	.000	.000	.035
Save money	75.7	62.1	59.3	.217	.000	.000
Buy delivery kit	17.8	6.3	12.2	.000	.000	.009
Arrange emergency transport	13.1	10.0	6.1	.003	.082	.000
Essential Newborn Care*						
Wiping baby with clean dry cloth	67.4	62.5	74.1			
Wrapping including head	24.6	13.8	18.2			
Cutting cord with sterilized thread	35.8	29.0	57.5			
Tying cord with sterilized thread	10.9	19.4	56.7			
Initiation of breastfeeding within 1 hour of birth	65.5	44.3	61.3	.000	.000	.325
Colostrums feeding	95.1	89.0	90.3	.596	.001	.003

*Multiple Response.

#### Antenatal care

No significant difference was observed between intervention and control areas for knowledge about ANC (P = 0.062). Men were well aware that advice for pregnant women regarding better dietary intake, resting in the day time, intake of iron folic acid and not doing heavy work should be given during ANC. This awareness existed across all study areas. Few men knew that advice on newborn care, family planning, birth preparedness and cell number of health worker should also be given during ANC. More than half of the respondents in the intervention knew about TT vaccination advice. Various clinical procedures were well known among the men as important during the ANC visit (Table 2).

#### Birth preparedness

Knowledge on saving money and determining attendant at delivery were significantly higher in intervention compared to control (p < 0.001). Although buying delivery kit and arranging emergency transport were still higher in the intervention than control, their levels remained low (17.8% and 13.1%, respectively) (Table 2).

#### Newborn care

Knowledge of men regarding wiping the newborn, cutting and tying the cord in a sterile manner were overall low, though comparatively higher in the control areas. Only knowledge of wrapping was higher in the intervention (Table 2). In the intervention, knowledge on initiation of breastfeeding within an hour, colostrum feeding, duration of exclusive breastfeeding, time of complementary food initiation, bathing of newborn after 3 days and shaving of hair after one month were higher (not all data shown).

#### Neonatal danger signs

One of the key activities of the IMNCS programme is to increase the knowledge of community members on neonatal danger signs. The male respondents were asked about their current knowledge on neonatal danger signs, the questions were spontaneous. More than 67% of the respondents of all study areas knew 1–2 neonatal danger signs; 24.8% of the respondents in the intervention were aware of 3–5 danger signs compared to 8.8% in control areas (Table 2).

#### Acute respiratory infection and diarrhoea of under-5 children

**Figure 1:**
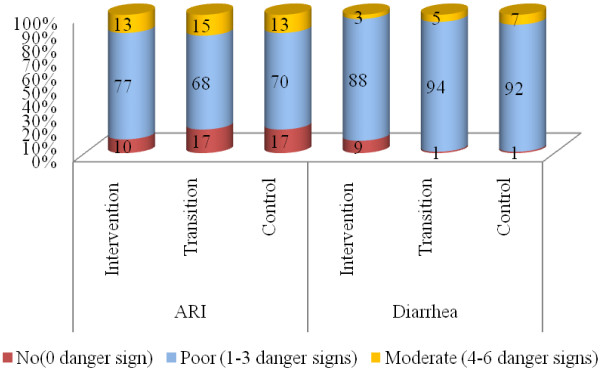
Knowledge on danger signs of ARI and Diarrhoea of under-5 children

Among the 10 danger signs of ARI promoted by the programme, no men could remember more than six danger signs. Most of them (70-77%) could remember 1–3 danger signs and 10-17% could remember none. In intervention, 9% of men had no knowledge of diarrhoeal danger signs compared to 1% in control areas. Most men had knowledge of 1–3 danger signs of diarrhoea (88-92%) (Figure 1).

Awareness on the use of oral rehydration therapy (ORT) during diarrhoea was universal. However, around one-third of the respondents were aware of the need of increased fluid intake during diarrhoea. Significantly more respondents in the intervention area were aware of the need to continue breastfeeding during diarrhoea (80.2% in intervention, 76.8% in transition and 70.1% in control areas) (data not shown).

### Men’s awareness of their wives’ maternal health care use

Men’s reports of their wives use of various services varied, with many reporting high ANC use by their wives and low experience of abortion (Table 3). This data cannot be interpreted by comparing intervention and control districts. This is discussed later under study limitations.

#### Decision-making

**Figure 2:**
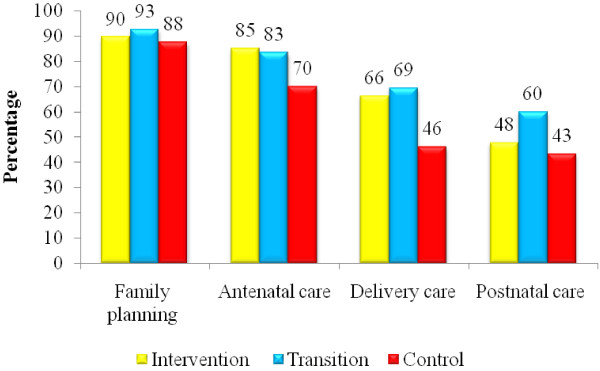
Joint decision-making with wives for various services

Most men reported joint decision-making with their wives regarding family planning. Fewer reported joint decision-making with regard to ANC, delivery and postnatal care. Joint decision-making was less common in the control areas for all types of care (Figure 2).

#### Transitional areas

Data from the transitional areas were included in the study because it acts as a proxy baseline in the absence of a baseline in our intervention district. In these areas, interventions were only in place for six months, so no changes resulting from the intervention were expected.

There were few differences in the background characteristics of the transitional areas compared with the other areas. In general, men in transition areas appeared to have less knowledge on maternal and neonatal care compared to the control. As expected, this knowledge was lower than that of the intervention. Regarding danger signs in children, the transitional area was similar to the control. In many indicators of men’s awareness of their wives’ use of maternal health care, transitional areas were lower than control. However, joint decision-making appeared higher in transitional compared to control areas and sometimes even in comparison with the intervention area.

## Discussion

This study aimed to identify the extent of men’s knowledge and awareness of MNCH issues between intervention and control districts and to ascertain if there were differences associated with the IMNCS intervention. We found that generally men’s knowledge and awareness was relatively high although there were few notable exceptions such as newborn care and birth preparedness.

It appears that IMNCS interventions are improving many aspects of men’s knowledge such as the content of antenatal care and the importance of determining birth attendant, provided that the interventions are of sufficient duration. We say this because the transition areas with only six months of exposure have not shown considerable changes compared to that of the intervention. An exception to the improvement in the intervention area is men’s knowledge of the appropriate age of conception for young women, as levels were lower in the intervention compared to the control group.

Antenatal care is an important determinant of safe delivery [31], and safe delivery is a proxy indicator for monitoring progress in maternal mortality [32]. Men’s knowledge regarding ANC (services and advice) in the intervention is almost universal. We cannot conclude though this level of knowledge was due to the presence of the IMNCS project, as we also noticed similar levels in control areas. Although certain obstetric emergencies cannot be predicted through antenatal screening, women as well as men can be educated to recognize and act on symptoms leading to potentially serious conditions [4,33]. In particular, the low levels of men’s knowledge of specific components of birth preparedness (buying delivery kits and arranging transport for emergency) is a concern and will need to be addressed as part of behaviour change communication.

Men’s knowledge on clean-birthing practices and keeping newborns warm was found poor. The control areas were better in some aspects of men’s knowledge on cord cutting and tying in sterile manner compared to intervention area. This may be due to better education and wealth status in some of the control areas [29] or due to other contextual factors such as NGOs (Sathi, Popy, Palli Shishu Foundation of Bangladesh, etc.) or projects working in the areas. The infrastructure may make these areas easier for government workers to access. However, these results imply the need for the IMNCS project to especially communicate newborn care messages to men. We also observed sub-optimal levels of knowledge of neonatal danger signs, danger signs of ARI and diarrhoea.

A greater proportion of men reported that they took decisions regarding MNCH issues jointly with their wives in intervention areas compared to that of control. We cannot come to the conclusion that IMNCS activities had an effect in this case because of the higher levels in the transitional areas. However, promoting joint decision-making in study settings is anticipated to be good practice.

Due to lack of baseline information it is not possible to make definite conclusions that our intervention had effect. The hypothesis that there should be no difference between control and intervention is however refuted by the differences that we did observe, suggesting possible changes resulting from IMNCS intervention.

Care is required in interpreting the findings of our study particularly those in Table 3. This table shows men’s reports of their wives’ reproductive health care practices. It may not be an accurate representation of women’s actual activities. So, we are unable to use these indicators to make a comparison between the intervention and control to determine effectiveness of IMNCS. Table 3 however does show that men may misreport their wives’ activities, for example, uptake of ANC is known to be higher than what men say. A separate study [29] provides women’s reporting of their own activities in relation to what their husbands said in our study. 

**Table 3:** Men’s awareness of their wives’ maternal health care use

	**Intervention**	**Transition**	**Control**	**P**	**P**	**P**
	**(1)**	**(2)**	**(3)**	**1 vs. 2**	**1 vs. 3**	**2 vs. 3**
N	959	2609	1979			
Use of FP method	71.3	67.5	70.3	.031	.582	.042
Experience of Abortion	12.5	14.4	17.8	.154	.000	.001
Experience of MR	4.1	4.3	3.5	.018	.009	.360
N	411	1032	793			
At least 1 ANC	82.0	56.3	72.8	.000	.000	.000
At least 4 ANC	38.2	9.3	21.4	.000	.000	.000
Delivery by medically trained provider	20.4	12.9	16.3	.000	.072	.041
Delivery by trained provider	61.6	34.2	46.9	.000	.000	.000
Received PNC within 48 hours from trained providers	35.5	7.8	8.7	.000	.000	.463

One of the challenges we faced was reaching men for interview during daytime. We did not reach our target sample, but we do not believe that this should change our interpretation of the results.

The retrospective nature of this study was another challenge which raises issues of recall bias, especially because some men were asked about events up to five years in the past. We instructed the enumerators to probe responses where necessary to reduce the recall bias.

## Conclusions

This study aimed to explore men’s knowledge on MNCH issues. Overall, men’s knowledge and awareness on older health promotion messages (use of modern FP method; what is diarrhoea, why the babies may experience it and what should be done during diarrhoea; receiving at least four ANCs from trained providers, etc.) was found better than newer messages (birth preparedness and newborn care). Nonetheless, the study provides evidence that men can learn and improve their awareness. With improved communication intervention a critical mass of men can be built up, who are aware of what can be done to improve women’s and children’s health particularly in relation to delivery, essential newborn and postpartum care.

This survey shows where men congregate for social interactions. Programme interventions should be directed to informal situations such as market places and tea stalls in order to reach as many men as possible. In response to these findings multimedia messages through television and radio could be utilized as these media are often available in such locations. In terms of the content of behaviour change communication messages, we conclude that deficiencies are likely to exist in men’s knowledge of two crucial and life saving components, birth preparedness and newborn care. The IMNCS programme recently introduced these components and we expect to see improvement in men’s knowledge in the future.

## Abbreviations

ANC, Antenatal Care; ARI, Acute Respiratory Infections; BCC, Behaviour Change Communications; FP, Family Planning; IMNCS, Improving Maternal, Neonatal and Child Survival; MNCH, Maternal, Neonatal and Child Health; MR, Menstrual Regulation; NGO, Non Government Organization; PNC, Postnatal Care; ORT, Oral Rehydration Therapy; SPSS, Statistical Packages for Social Sciences; TT, Tetanus Toxoid.

## Competing interests

The authors declare that they have no competing interests.

## Authors’ contributions

HEN was the principle investigator of the study and primarily conceptualized the research. HEN, ML and PN participated in the planning and conception of the research questions and the study design. HEN and PN were responsible for analyzing the data. HEN and ML drafted the article and critically revising the manuscript for important intellectual content. All authors gave suggestions, read manuscript carefully, fully agreed on its content and approved its final version.
